# Supplementary material for: U-shaped association between waist-to-height ratio and microalbuminuria: A cross-sectional analysis conducted within the Chinese demographic
Source: PLoS One. 2026 May 19;21(5):e0349370. doi: 10.1371/journal.pone.0349370 (PMC13186341; doi:10.1371/journal.pone.0349370)
Supplement: S1 Table — This table presents the variance inflation factor (VIF) values for all covariates included in the multivariable models. (DOCX) [file pone.0349370.s001.docx]

**Table S1. Collinearity diagnostics steps.**

| Variable | VIF  Step 1 |
| --- | --- |
|  |  |
| Gender | 1.9 |
| Smoking status | 1.5 |
| Drinking status | 1.3 |
| ALT(U/L) | 3.1 |
| AST(U/L) | 3 |
| eGFR (ml/(min×1.73m2) | 4 |
| SBP (mmHg) | 1.9 |
| DBP (mmHg) | 1.8 |
| Weight(kg) | 2.9 |
| HC (cm) | 2.4 |
| HDL-c(mmol/L) | 1.5 |
| LDL-c(mmol/L) | 1.2 |
| TG (mmol/L) | 1.2 |
| FPG (mmol/L) | 1.1 |
| Tumor history | 1 |

SBP, systolic blood pressure; DBP, diastolic blood pressure; eGFR, estimated glomerular filtration rate; AST, aspartate aminotransferase; ALT, alanine aminotransferase; HDL-c, High-density lipoprotein cholesterol; LDL-c, Low-density lipoprotein cholesterol; HC, Hip circumference; TG, Triglyceride; FPG, Fasting plasma glucose.

Abbreviation: VIF: variance inflation factor; VIF = 1/(1-R2).

Note: The variables with VIF>5 will be regarded as collinear variables and cannot be included in the multiple regression model.
