# Supplementary material for: U-shaped association between waist-to-height ratio and microalbuminuria: A cross-sectional analysis conducted within the Chinese demographic
Source: PLoS One. 2026 May 19;21(5):e0349370. doi: 10.1371/journal.pone.0349370 (PMC13186341; doi:10.1371/journal.pone.0349370)
Supplement: S2 Table — This table compares demographic, anthropometric, biochemical, and clinical variables between participants with WHtR < 0.497 (n = 8,643) and those with WHtR ≥ 0.497 (n = 25,042). (DOCX) [file pone.0349370.s002.docx]

**Table S2. The characteristics of participants on both sides of the inflection point.**

| WHtR | < 0.497 | ≥ 0.497 | P-value |
| --- | --- | --- | --- |
| Participants | 8643 | 25042 |  |
| WC (cm) | 84.88 ± 5.44 | 89.94 ± 7.70 | < 0.001 |
| HC (cm) | 90.63 ± 5.74 | 99.13 ± 7.14 | < 0.001 |
| Weight(kg) | 56.18 ± 8.51 | 65.95 ± 10.64 | < 0.001 |
| Height(cm) | 161.55 ± 7.48 | 160.01 ±8.09 | < 0.001 |
| Scr (umol/L) | 67.89 ± 18.52 | 68.77 ± 16.38 | < 0.001 |
| HDL-c(mmol/L) | 1.41 ± 0.36 | 1.28 ± 0.32 | < 0.001 |
| LDL-c (mmol/L) | 2.83 ± 0.87 | 3.03 ± 0.90 | < 0.001 |
| Chol (mmol/L) | 4.91 ± 1.11 | 5.13 ± 1.13 | < 0.001 |
| TG (mmol/L) | 1.32 ± 0.89 | 1.80 ± 1.27 | < 0.001 |
| ALT (U/L) | 13.00 (10.00-18.00) | 15.00 (11.00-22.00) | < 0.001 |
| AST (U/L) | 20.00 (16.00-24.00) | 20.00 (17.00-25.00) | < 0.001 |
| γ-GGT (U/L) | 17.00 (13.00-25.00) | 22.00 (16.00-34.00) | < 0.001 |
| FPG (mmol/L) | 5.63 ± 1.44 | 6.11 ± 1.79 | < 0.001 |
| PPG (mmol/L) | 7.43 ± 3.32 | 8.90 ± 4.09 | < 0.001 |
| HbA1C (%) | 5.87 ± 0.89 | 6.17 ± 1.09 | < 0.001 |
| BMI(kg/m^2^) | 21.46 ± 2.49 | 25.68 ± 3.15 | < 0.001 |
| eGFR (ml/(min×1.73m^2^) | 95.78 ± 19.31 | 93.35 ± 18.94 | < 0.001 |
| ACR (mg/g) | 9.23 (546-17.80) | 10.24 (5.85-20.72) | 0.024 |
| AGE (years) | 56.74 ± 9.37 | 58.32 ± 9.18 | < 0.001 |
| SBP (mmHg) | 122.81 ± 17.82 | 134.89 ± 20.29 | < 0.001 |
| DBP (mmHg) | 74.25 ± 10.20 | 78.73 ± 10.83 | < 0.001 |
| Tumor |  |  | 0.752 |
| yes | 257 (2.97%) | 728 (2.91%) |  |
| no | 8386 (97.03%) | 24314 (97.09%) |  |
| Sex |  |  | 0.113 |
| Male | 2806 (32.47%) | 8363 (33.40%) |  |
| Female | 5837 (67.53%) | 16679 (66.60%) |  |
| Smoking |  |  | 0.002 |
| no | 7161 (83.67%) | 21165 (85.19%) |  |
| yes | 1398 (16.33%) | 3680 (14.81%) |  |
| Drinking |  |  | < 0.001 |
| no | 6166 (71.99%) | 18526 (74.54%) |  |
| yes | 2409 (28.01%) | 6329 (25.46%) |  |
| Obesity |  |  | <0.001 |
| no | 8590 (99.39%) | 20109 (80.30%) |  |
| yes | 53 (0.61%) | 4933 (19.70%) |  |
| Diabetes Mellitus |  |  | <0.001 |
| no | 7490 (86.66%) | 18551 (74.08%) |  |
| yes | 1153 (13.34%) | 6491 (25.92%) |  |
| Microalbuminuria |  |  | <0.001 |
| no | 8595 (99.44%) | 24786 (98.98%) |  |
| yes | 48 (0.56%) | 256 (1.02%) |  |

Values are expressed as N (%), mean ± SD or medians (quartiles)
