# Supplementary material for: U-shaped association between waist-to-height ratio and microalbuminuria: A cross-sectional analysis conducted within the Chinese demographic
Source: PLoS One. 2026 May 19;21(5):e0349370. doi: 10.1371/journal.pone.0349370 (PMC13186341; doi:10.1371/journal.pone.0349370)
Supplement: Attachment — Submitted filename: Response to Reviewers.docx [file pone.0349370.s005.docx]

Dear Editor and Reviewers,

Thank you very much for giving us the opportunity to revise our manuscript entitled “U-shaped Association Between Waist-to-Height Ratio and Microalbuminuria: A Cross-Sectional Analysis Conducted Within the Chinese Demographic” (Manuscript ID: PONE-D-25-55420). We greatly appreciate the reviewers’ thoughtful and constructive comments, which have been very helpful in improving the quality of our paper. We have carefully revised the manuscript according to all the comments.

In addition, we would like to provide several clarifications and corrections regarding our initial response letter, as we identified a few points that were incomplete or not sufficiently precise upon further review guided by our institutional supervisor. Below is our point-by-point response to each comment from the editorial office and the reviewers. All changes in the revised manuscript are highlighted in yellow for your easy reference.

Thank you and best regards.

Yours sincerely,

*Corresponding author

Heping Zhang，

Department of Nephrology, Affiliated Hospital of North Sichuan Medical College,

No.1 Maoyuan South Rd,

Nanchong 637000,

Sichuan Province,

China.

E-mail：867801115@qq.com;

*Corresponding author

Yongcheng He，

Department of Nephrology, Affiliated Hospital of North Sichuan Medical College,

No.1 Maoyuan South Rd,

Nanchong 637000,

Sichuan Province,

China.

Email: heyongcheng640815@126.com

We have also addressed all technical requirements, including data deposition and manuscript formatting, as detailed below.

Response to Editorial Office’s Requirements:

Comment 1 (from Editor): Please ensure that your manuscript meets PLOS ONE's style requirements...

Response: Thank you for the reminder. We have carefully reviewed the PLOS ONE formatting template and have adjusted our manuscript’s formatting accordingly (e.g., file naming, title page layout, etc.) to ensure it meets the journal’s standards.

Comment 2 (from Editor): Thank you for stating the following financial disclosure... Please state what role the funders took in the study.

Response: We thank the editor for pointing out this omission. We confirm that this study was supported by the Shenzhen Science and Technology Innovation Committee (Grant No. JCYJ20210324133412033) and the Shenzhen Municipal Health Commission (Grant No. SZXJ2017031). The funders provided financial support for the research but had no role in study design, data collection and analysis, decision to publish, or preparation of the manuscript. We have now clearly stated this in the revised manuscript and trust that the editorial office will update the online submission form accordingly.

Comment 3. Please update your submission to use the PLOS LaTeX template. The template and more information on our requirements for LaTeX submissions can be found at http://journals.plos.org/plosone/s/latex.

Response: We thank the editor for the suggestion. However, our manuscript was prepared using Microsoft Word, not LaTeX. We have carefully followed the PLOS ONE formatting templates for Word submissions and ensured that all style requirements (e.g., title page, file naming, reference format) are met. We believe the current Word-based manuscript is correctly formatted for the journal's system. Please let us know if any further adjustments are needed.

Comment 4 (from Editor): ...the repository you have noted... does not qualify as an acceptable data repository...

Response: We thank the editor for pointing this out. To comply with PLOS’s data policy, we have now deposited our minimal dataset in figshare and it can be accessed via the following DOI: 10.6084/m9.figshare.31404420. We have updated the Data Availability Statement in the manuscript accordingly.

Additionally, we have carefully revised the entire manuscript to remove any incorrect references to the “PLOS ONE database” (e.g., in the “Data source” and “Supplementary Information” sections) to ensure consistency with our figshare deposition. All changes are highlighted in yellow.

Comment 5. Please ensure that you include a title page within your main document. You should list all authors and all affiliations as per our author instructions and clearly indicate the corresponding author.

Response: Thank you for the reminder. We have now ensured that a title page is included within the main manuscript document. The title page lists all authors and their affiliations in accordance with PLOS ONE's author instructions, and the corresponding author is clearly indicated with an asterisk (*) and a corresponding footnote. This can be found on Page 1 of the revised manuscript.

Comment 6. Please upload a copy of Supporting Information Figure/Table/etc. which you refer to in your text on page 31.

Response: Thank you for pointing this out. After carefully reviewing page 31 of our manuscript, we confirm that the item referenced there is Table 5, which is part of the main manuscript text and has been properly embedded. Therefore, no additional Supporting Information file is required specifically for the reference on page 31.

Separately, we noticed that Table S1 is referenced on page 10 but was inadvertently omitted from the original submission. We have now prepared and uploaded this Supporting Information file as TableS1.docx. The file contains the results of multicollinearity assessment, showing variance inflation factor (VIF) values for all independent variables included in the regression models, confirming that no substantial collinearity (VIF > 5) was present. The file has been formatted according to PLOS ONE's guidelines. It is uploaded as a separate file in the submission system.

We apologize for the omission and have ensured that all supporting information files are now correctly provided. Please let us know if any further adjustments are needed.

Comment 7. If the reviewer comments include a recommendation to cite specific previously published works, please review and evaluate these publications to determine whether they are relevant and should be cited. There is no requirement to cite these works unless the editor has indicated otherwise.

Response: We have carefully reviewed all comments from both reviewers and confirm that no specific previously published works were recommended for citation. Therefore, no additional references were added based on this requirement. We will, of course, be happy to consider any specific suggestions if the editor deems them necessary.

Comment 8. Please review your reference list to ensure that it is complete and correct. If you have cited papers that have been retracted, please include the rationale for doing so in the manuscript text, or remove these references and replace them with relevant current references. Any changes to the reference list should be mentioned in the rebuttal letter that accompanies your revised manuscript. If you need to cite a retracted article, indicate the article’s retracted status in the References list and also include a citation and full reference for the retraction notice.

Response: Thank you for your valuable comments on our manuscript. We have carefully reviewed and revised the reference list according to your suggestions.

The following modifications have been made:

Reference [6] has been updated by adding the URL access information as required.

References [12] and [17] have been reformatted to comply with the PLOS ONE reference style guidelines.

Upon verification, we found that references [9], [10], [13], [16], [37], and [38] could not be accessed through standard academic databases, suggesting they may have been retracted or are no longer available. Therefore, we have replaced these with new, accessible and relevant references to support the corresponding statements in our manuscript. All new references are current and appropriately cited.

All changes to the reference list have been clearly indicated in the revised manuscript and are summarized in the point-by-point response provided in the rebuttal letter accompanying this revision. We believe these revisions have strengthened the manuscript and ensured the accuracy and completeness of our references. We thank you again for your guidance and hope that the revised version meets the requirements for publication.

**Response to Reviewer #1:**

Comment: The article is well written... However, the area that needs little touch is the discussion section. The Authors quoted a lot of values in the discussion section... I recommend that the authors review the discussion section to remove all the "confidence intervals" and all the values that can be removed from the section.

Response: We sincerely thank the reviewer for this positive and constructive feedback.

Following this valuable suggestion, we have thoroughly revised the “Discussion” section. We have removed most of the detailed statistical values (such as specific odds ratios and confidence intervals) and focused more on the interpretation of our findings, the underlying mechanisms, and comparisons with previous literature. We believe this has made the discussion more concise and reader-friendly. The revised discussion can be found on (Pages 16-19).

Response to Reviewer #2:

Comment 1: ...elaborating more on the physiological mechanisms that explain the U-shaped relationship.

Response: We thank the reviewer for this insightful suggestion. In response, we have expanded the Discussion section to provide a more in-depth exploration of the potential physiological mechanisms underlying the U-shaped association.

Specifically, we have added content in the Discussion section (Page 18) discussing that at the low end of WHtR, the increased risk of microalbuminuria might be related to sarcopenia or chronic undernutrition, which can lead to systemic inflammation and endothelial dysfunction. At the high end of WHtR, we further elaborated on the classical pathways, including insulin resistance, activation of the renin-angiotensin-aldosterone system (RAAS), and adipokine imbalance, which are known to contribute to glomerular damage. We hope this addition addresses the reviewer's concern.

Comment 2: ...could raise concerns regarding redundancy if the data have already been heavily analyzed elsewhere. The authors should explicitly compare their findings with previous studies that used similar datasets...

Response: We appreciate the reviewer’s attention to this important issue. To clarify the novelty of our work, we have added a statement in the “Introduction” section (Page 4). We now explicitly state: “While the REACTION study (Risk Evaluation of cAncers in Chinese diabeTic Individuals: a lONgitudinal study) has been used in previous epidemiological studies, to the best of our knowledge, this is the first investigation specifically focusing on the non-linear, U-shaped relationship between waist-to-height ratio and microalbuminuria. Previous research has primarily examined linear associations or other obesity indices, making our study a unique contribution to the field.”

Comment 3: ...the cross-sectional nature of the study limits the ability to infer causality... discuss the limitations... more prominently...

Response: We agree completely with the reviewer. We have now strengthened the language regarding this limitation. In the “Discussion” section (Page 19), we have revised the text to read: “Most importantly, the cross-sectional design of this study inherently precludes any causal inferences. While we identified a robust U-shaped association, we cannot determine whether a low or high WHtR leads to microalbuminuria, or whether reverse causality or unmeasured confounding explains this relationship.” We have also added a sentence in the “Conclusion” section (Page 20) explicitly calling for future longitudinal cohort studies to validate our findings and establish causality.

Comment 4: ...handling of missing data is not explicitly mentioned... provide further justification for the sample size...

Response: We thank the reviewer for raising these methodological points. We have now clarified these issues in the revised manuscript (Methods section, Page 12).

Regarding missing data: In our study, there were no missing values for the key variables (WHtR, UACR) after applying the exclusion criteria. For categorical covariates with missing data (e.g., smoking and drinking status), referring to recent literature (e.g., Liao et al., 2025), we retained these participants and classified them as a separate category ("Not recorded") rather than excluding them from the analyses. This approach optimizes statistical power and minimizes potential selection bias. No imputation was performed.

Regarding sample size justification: We performed a post-hoc power analysis to confirm that our sample size was sufficient to detect the observed associations. The actual observed prevalence of microalbuminuria in our study population was 14.4%. Based on this, the study had >99% power to detect an odds ratio of 1.2 for microalbuminuria at a two-sided alpha level of 0.05. This high statistical power supports the robustness of our findings. We have added this information to the Methods section.

We sincerely apologize for any confusion or inconvenience caused by the omissions or inaccuracies in our initial response. We are fully committed to ensuring the highest standards of transparency and scientific rigor in our work. We hope these clarifications and revisions are acceptable, and we kindly request that the revised manuscript and response letter be reconsidered in light of these updates.

We have also uploaded the revised manuscript (with changes highlighted) as a separate file in the submission system for your reference.

Thank you very much for your time and understanding. Please do not hesitate to contact us if any further information is needed.

Yours sincerely,

On behalf of all authors,

Heping Zhang, MD

Yongcheng He, MD
